# Experiences of Patients After Withdrawal From Cancer Clinical Trials

**DOI:** 10.1001/jamanetworkopen.2021.20052

**Published:** 2021-08-10

**Authors:** Connie M. Ulrich, Kathleen Knafl, Anessa M. Foxwell, Qiuping Zhou, Cynthia Paidipati, Deborah Tiller, Sarah J. Ratcliffe, Gwenyth R. Wallen, Therese S. Richmond, Mary Naylor, Thomas F. Gordon, Christine Grady, Victoria Miller

**Affiliations:** 1 School of Nursing, University of Pennsylvania, Philadelphia; 2 Perelman School of Medicine, University of Pennsylvania, Philadelphia; 3 School of Nursing, University of North Carolina, Chapel Hill; 4 School of Nursing, George Washington University, Washington, DC; 5 Department of Family and Community Health, Marcella Niehoff School of Nursing, Loyola University Chicago, Chicago, Illinois; 6 Public Health Sciences, University of Virginia, Charlottesville; 7 National Institutes of Health Clinical Center, Bethesda, Maryland; 8 Department of Psychology, University of Massachusetts, Lowell; 9 Department of Pediatrics, Children’s Hospital of Philadelphia, Philadelphia, Pennsylvania

## Abstract

**Importance:**

Cancer clinical trials (CCTs) provide patients an opportunity to receive experimental drugs, tests, and/or procedures that can lead to remission. For some, a CCT may seem like their only option. Little is known about experiences of patient-participants who withdraw or are withdrawn from CCTs.

**Objective:**

To examine patient-participants’ experiences during withdrawal from CCTs.

**Design, Setting, and Participants:**

This qualitative, descriptive study used a semistructured interview designed specifically for it, with open-ended and probing questions. The study took place at a National Cancer Institute–designated comprehensive cancer center affiliated with the University of Pennsylvania. The need for a sample of 20 interviewees was determined by code and meaning saturation (ie, no new themes revealed and identified themes fully elaborated). Interviews were transcribed verbatim and analyzed with a qualitative software program. Data coded with the software were refined into categories reflecting broad themes. A criterion-based sampling approach was used to select a subset of adult patients with cancer who were former CCT participants and who agreed on exit from those CCTs to a later interview about withdrawal experiences. They were contacted one by one by telephone from September 2015 through June 2019 until 20 agreed. Data analysis was completed in October 2020.

**Main Outcomes and Measures:**

Themes characterizing patient-participants’ perceptions of their withdrawal experiences.

**Results:**

Respondents’ mean (SD) age was 64.42 (8.49) years; 12 (63.2%) were men. Most respondents were White (18 respondents [94.7%]) and college educated (11 respondents [55.0%]). Cancer stage data were available for 17 participants, 11 of whom (64.7%) had stage IV cancer at CCT enrollment. Thirteen respondents reported withdrawal as a result of disease progression, and 5 withdrew because of adverse effects. Other reasons for withdrawal included acute illness and participant uncertainty about the reason. Analysis of interview data yielded 5 themes: posttrial prognostic awareness, goals of care discussions, emotional coping, burden of adverse effects, and professional trust and support. Subthemes included regrets or hindsight, urgency to start next treatment, and weighing benefits and burdens of treatment. Limited discussions about patient-participants’ immediate posttrial care needs left many feeling that there was no clear path forward.

**Conclusions and Relevance:**

Patient-participants transitioning from a CCT described feeling intense symptoms and emotions and awareness that their life span was short and options seemed to be limited. Communication that includes attention to posttrial needs is needed throughout the CCT to help patient-participants navigate posttrial steps. Research should focus on components of responsible and ethical CCT transitions, including types and timing of discussions and who should begin these discussions with patient-participants and their families.

## Key Points

**Question:**

What are the experiences of patient-participants when they leave a cancer clinical trial?

**Findings:**

In this qualitative study in which 20 patients with cancer who withdrew or were withdrawn from clinical trials were interviewed, 5 themes emerged: posttrial prognostic awareness, goals of care discussions, emotional coping, burden of adverse effects, and professional trust and support.

**Meaning:**

Clinical trial exit is a critical time for engaging patients and families, who have invested their time and hopes in the trial, in meaningful dialogue about posttrial care needs; ongoing research is needed to inform responsible transitions from clinical trials and ensure ethical treatment of patient-participants.

## Teaser

This qualitative study examines patient-participants’ experiences during withdrawal from cancer clinical trials.

## Introduction

Patients with cancer participate in research trials for many reasons, including hope for remission or cure or a desire to give something back to society.^1,2^ Participation may be viewed as the only option available. We know little, however, about the experiences of patients with cancer when they withdraw or are withdrawn from cancer clinical trials (CCTs) and confront the possibility of limited life expectancy.

Transitions occur every day in health care as patients move between clinical units, acute and primary care systems, and various health care practitioners. Transitions are especially vulnerable times for at-risk patients, many of whom are experiencing deteriorating health status and must cope with changes in care delivery. Patients who withdraw or are withdrawn from CCTs represent such a patient group. Transitional care is operationally defined as a time-limited range of services designed to enable at-risk patients to modify their goals and optimize their quality of life.^3,4^ Such services are essential for high-quality outcomes among patients who withdraw or are withdrawn from CCTs. Transitions can create uncertainty and confusion for patients and families, with the potential for miscommunication, medical errors, and poor outcomes. As Ulrich et al^5^ have argued, “Patients who complete a CCT may be feeling well but frightened of disease recurrence and unsure of future treatment plans.” When patients are nearing the end of their lives and previous treatments have failed, CCT transitions can be very difficult. Effective transitional care is achieved by providing such patients and their family caregivers with access to a trusted clinician who will ensure continuity of care, prevent avoidable and costly breakdowns in care, and advocate to ensure that patients achieve what matters most to them. Communication processes between patient-participants and practitioners at CCT transitions are not well understood, nor is the association between poorly managed transitions and subsequent patient morbidity.

The voices of patient-participants who have exited CCTs are often missing from research ethics literature. Dresser^6^ pointed out that most research ethics reports incorporate perspectives of ethicists, clinicians, and researchers, not those of patients who participate in CCTs. Also missing are family members’ perspectives. There are many gaps in our understanding of patient-participants’ withdrawal from CCTs, including reasons for trial exit, supports received during the transition, and planning for post-CCT next steps and treatment options. Closing these gaps can inform development of evidence-based guidelines for ethical practice and informed decision-making during trial exits. Therefore, the purpose of this qualitative study was to further our understanding of patients’ experiences during and after trial exit by interviewing CCT patient-participants who withdrew or were withdrawn from CCTs.

## Methods

### Sample and Design

Criterion-based sampling was used to select patient-participant cases meeting a certain criterion (in this case withdrawal from CCTs).^7^ As such, participants in this study were a subset of 498 adult CCT patient-participants who consented between September 2015 through June 2019 to a sequential mixed-methods parent study of their views about research participation and retention in CCTs at a Northeast urban National Cancer Institute–designated comprehensive cancer center. Patient-participants were aged 18 years or older, with a cancer diagnosis in the following categories: gastrointestinal or genitourinary cancer, hematological or lymphatic malignant disorders, lung cancer, and breast or gynecological cancer. Of the 498 who consented to the parent study, 20 who had withdrawn or been withdrawn from their CCTs and who indicated a willingness to be subsequently interviewed were contacted for a semistructured interview about their withdrawal experiences. Nineteen also completed a baseline survey about their CCT experience. Patient-participants were provided with a nominal incentive ($20) for their participation. Verbal informed consent was obtained from all participants by telephone. This study follows the Consolidated Criteria for Reporting Qualitative Research (COREQ) reporting guideline to promote transparency and quality of the research process.^8^ The study was approved by the institutional review board at the University of Pennsylvania and the affiliated cancer center.

### Data Collection

Sociodemographic information was collected as part of the larger study, and an interview script of open-ended questions (semistructured interview) was developed by the research team. Race/ethnicity was self-reported by the patients. Race/ethnicity was assessed in this study because disparities continue to exist in CCT research participation. We used an interview guide that balanced structure with flexibility, and all patient-participants were asked the same open-ended questions and encouraged to discuss their perspectives and experiences related to each question. Probing (follow-up) questions were used to gain further insights and more detailed accounts of experiences.^9,10^ Interview scheduling was flexible and was based on patient-participants’ preferences. Interviews were conducted by 2 postdoctoral fellows (C.P. and another fellow who is not a coauthor of this article) working with the principal investigator (C.U.), all of whom were academic researchers with a clinical background. All participants were willing to share their perspectives with the research team. When challenging topics or emotions arose, the interviewers used therapeutic communication to support participants. Interviews were audiotaped, transcribed verbatim, uploaded to a secure site, and transferred to NVivo qualitative software version 12 (SQR International). On the basis of our previous qualitative research with a similar study population, we anticipated needing 20 to 25 interviews to identify and fully develop themes. We achieved saturation at 20 interviews when the interviews no longer revealed new themes (code saturation) and identified themes were fully elaborated (meaning saturation).^9^

### Statistical Analysis

We used descriptive statistics for demographic and clinical characteristics, including age, sex, education, and cancer type. We used principles of conventional content and thematic analysis to explore patient-participants’ CCT withdrawal experiences.^11,12^ Transcripts were initially reviewed by team members with qualitative expertise (C.U., K.K., and A.M.F.), who coded phrases, sentences, and paragraphs. The list reflected both preset codes based on interview guide questions (eg, benefits and burdens of CCT participation) and codes inductively derived from the interview transcripts. NVivo was used to code interviews and access data on selected codes. Coded data were reviewed and refined into categories reflecting broader themes characterizing withdrawal experiences (described in the Results). Data analysis was performed using NVivo and Stata SE statistical software version 16 (StataCorp) and was completed in October 2020.

## Results

### Sample Characteristics

The sample consisted of 20 patient-participants who agreed to be interviewed about their CCT withdrawal; 19 had provided baseline data (Table 1). Respondents’ mean (SD) age was 64.42 (8.49) years. The sample consisted of more men (12 men [63.2%]) than women (7 women [36.8%]); information on sex was missing for 1 respondent. Most respondents were White (18 respondents [94.7%]) and college educated (11 respondents [55.0%]). Cancer stage data were available for 17 participants, 11 of whom (64.7%) had stage IV cancer at CCT enrollment; 10 of 20 participants (50.0%) had stage IV cancer at diagnosis. Thirteen participants reported being withdrawn because of disease progression, 5 were withdrawn because of adverse effects, 1 was withdrawn because of acute illness, and 1 patient-participant was uncertain as to the reason for withdrawal (Table 2).

**Table 1.** Sample Characteristics

Characteristic	Participants, No. (%) (N = 20)
Age, mean (SD), y	64.42 (8.49)
Sex^a^	
Female	7 (36.8)
Male	12 (63.2)
Race^a^	
White	18 (94.7)
Black or African American	1 (5.3)
Hispanic or Latino ethnicity	1 (5.3)
Education	
Less than a high school degree	0
High school graduate	3 (15.0)
Some college	6 (30.0)
College graduate	6 (30.0)
Postgraduate degree	5 (25.0)
Time from trial consent to withdrawal from trial, mean (SD), d	143 (95.3)
Cancer stage at enrollment in cancer clinical trial^b^	
II	2 (11.8)
III	4 (23.5)
IV	11 (64.7)
Eastern Cooperative Oncology Group scale score^c^	
0	9 (45.0)
1	11 (55.0)

^a^ Data are missing for 1 participant.

^b^ Data are missing or not available for 3 participants.

^c^ On scale for assessment of disease progression, 0 indicates fully active, able to carry on all predisease performance without restriction, and 1 indicates restricted in physically strenuous activity but ambulatory and able to carry out work of a light or sedentary nature (eg, light housework or office work).

**Table 2.** Patient-Participant–Stated Reason for Withdrawal, With Illustrative Quotations

Reason	Decided by	Quotation
Progression of disease (n = 13)	Clinical trial protocol	“But unfortunately, after six months I had a tumor that decided it was going to grow through my chest wall.” “Other that the fact it wasn’t working for me? That was…it wasn’t… according to him, the sharp increase in the white blood cells was semi-critical enough that he put me in the hospital for four days.” “I stopped treatment there because I had progression of disease, so I was, you know, taken out.” “Unfortunately, developing metastasis in the bone made me no longer eligible for the clinical trial.” “I had no side effects, and I couldn’t have been happier. My doctor was very happy. Then what happened was I had a scan and the scan came back saying that I had one or two new nodes that were somewhat active in my neck, as I recall. The protocol of the trial says any new nodes, you know, you come off the trial. Well, that’s where the protocol of the trial and the patient benefit collided because I’m obviously benefiting if their own records show that I had a 70% reduction and I had no side effects and no nodes that I could feel.”
Adverse effects (n = 5)	Physician (2 oncologists and 1 neurologist)	“It was a matter of let’s stop this now because it’s only gonna get worse. And the interesting part about this was even though it inflamed my lungs, um, the oncologist said it was working…that’s why we…I withdrew and everybody like came to the consensus that it was best…At that specific time he said let’s leave the door open like for…you know, may be down the road you’d want to, you know, start this again.” “I went for the neurologist follow-up in two weeks she said ‘I’m sorry, you can no longer take that medicine.’”
	1 Patient decision and 1 joint decision	“When I saw the doctor, I said I’m getting off of it…Their face went wide open, like, why you getting off of it?” “Uh, no, it was me. I was like, I’m never taking that drug again [laughing]. No, no. Actually, but he [oncologist]…yeah, he absolutely agreed. He’s like, absolutely you’re not going to take it again.”
Acute illness (n = 1)	Clinical trial protocol (antibiotic was contraindicated)	“Um…[mumbling] um…no, I, um…I’m trying to think back that far…What I did with that. Um…what did I stop it for? What did I stop it for? Maybe it was because…yeah, I had to go in the hospital. I had an ear infection…really serious ear infection and my ear drum…ear drum burst or whatever.”
Uncertain (n = 1)	Researcher	“I mean they don’t tell you why you were taken off. They just took you off. So [inaudible] a clinical trial…all the people were on…‘cause we’re only given numbers, no names or anything. And, uh, we were just taken off it, so I just assume it didn’t work.”

### Qualitative Themes

Five major themes emerged: post-CCT prognostic awareness, goals of care discussions, emotional coping, burden of adverse effects, and professional trust and support. In addition, some subthemes were identified, including regrets or hindsight, urgency to start next treatment, hope, the odds of effectiveness or survival, trade-offs, and weighing benefits and burdens of treatment. Transcripts documented patient-participants’ unique cancer journeys, disappointment regarding a failed trial, and examination of personal values after withdrawal. Themes and subthemes are discussed later in the article. Table 3 presents illustrative quotations.

**Table 3.** Themes, Subthemes, and Illustrative Quotations

Theme and subtheme	Quotation
Posttrial prognostic awareness	“I stopped treatment there because I had progression of disease so I was, you know, taken out.” “The trial stopped working.” “I mean they don’t tell you why you were taken off. They just took you off. So [inaudible] a clinical trial…all the people were on…‘cause we’re only given numbers, no names or anything. And, uh, we were just taken off it, so I just assume it didn’t work.” “So, he said we’re at a balancing thing here that, you know, we’re not controlling the disease and we’re not giving you the drugs that we could give you to help you feel better, so therefore, you know…and my recommendation is, you know, go off the trial, and I agreed. You know?”
Where the patient is now	“I’m doing reasonably well. I left the [hospital] study because I had a recurrence. And so obviously, I’m not doing great, but I’m doing reasonably well.” “So now I’m in a hospital bed and I can’t walk, even with…even with a walker.” “Um, the first two therapies, the one that was the trial failed and the one after that, I found out yesterday, also failed.” “Horrible right now.” “So unfortunately, it was, uh…it was beyond the scope of the clinical trial, essentially within a 20 percent increase and, uh, it’s not much. It’s only like about two millimeters or something, but still it’s…the threshold is 20 percent, so they had to take me out of that trial, but I’m starting a new one on this Monday.” “So that’s where I’m at. Right now, I’m no longer in treatment. I am actually in hospice care right now.”
Regrets, hindsight, or should-haves	“Well, since they removed me from the trial which incidentally, I did not agree with.” “You know, I no longer qualified, so in one respect, you know, that wasn’t…I had no choice.” “But, you know, the next six months of my…you know, I probably can’t fly the way I want to and travel the way I want to and…This is kind of messing the rest of my life up…I’m frustrated and angry, to be quite honest with you—because…this is not the summer the way I thought. Okay, I’m done my treatment. I’m gonna be fine. I was feeling good. And I started taking this stupid drug and all of a sudden, you know, I’ve had the reaction and now the PEs, and now the next six months or whatever, it’s gonna be I have to stay on blood thinners and…you know, I can’t go back to karate. Like all the things that make me sane, I can’t do.” “So, you know, there was some kind of…maybe had I not gone onto the study and maybe had I gone onto a medication that they knew had certain…you know. It was all that kind of thoughts in my mind regarding it, but we don’t know. It was a good, um…thought-out process for me to be in the study [inaudible]. So consequently, you know, we did what we thought was best at the time.”
Urgency to start next treatment	“And, ‘cause that’s the criteria they use and that’s the way it turned out unfortunately. But, so then we immediately started talking about, uh…about other options and began as always. He said I want straight chemo. Um, this trial that I’m going to go in, which by the way, doesn’t even have a name yet. There just a number. It’s like [Name], something or other. It’s like a seven-digit number.” “But we’re trying for another one. If that doesn’t work, we’ll try another one. We’ll keep going till we get it right.”
Goals of care discussions	
Decision to enroll or other options	“I didn’t participate in it because I was this clean-cut American that wanted to contribute to all cancer research. I mean, that was a side benefit. I was happy to do that, but I was on it mainly for selfish reasons is because they had no approved drugs for me that were left. That’s why Doctor [Name] put me on me a trial.” “They saved my life the first time. My thoughts were, they’ll save my life a second time…And I have more at stake now ‘cause I have two kids, you know?...So now it’s even more important that I’m okay.” “That’s what put me into the clinical trial, so yeah, that is what kept me in the clinical trial, too, is that, you know, we were hoping that it would be the drug that we needed to unlock the key.”
Living with cancer	“So, there were numerous CAT scans, and so those ups and downs are difficult. You know, you hope for the best and that sort of thing. So…you know, that’s just part of…part of living with cancer.”
The odds of effectiveness or survival and trade-offs	“There’s risks, I understand that…but it’s acceptable risk. It’s worth the, uh…when you balance…I guess like the doctors do, they balance, you know, the good part against the risk. And it’s the right thing to do and I try not to think too much about the downside.” “I mean when you have one lung and you’re seventy-eight years old, you grab at the straws. That’s the way I feel. If there’s a chance, I’m going to do it.” “The type of cancer I have, the only cure is a bone marrow transplant and it’s a 40 percent chance that it might work and the rest is that it either comes back or it kills you.” “I would weigh it in the same respect as I weighed this one. Okay? What are the statistical chances of success? If there is any success, what are the benefits, remission or slowing of the progression of the disease and what not? What are the potential side effects, what not, so there are side effects? I wanted to take all of those into consideration, disruption of your life. When doing this other trial, you know when you go up there, you’d be up there 12 hours.”
Not just a number	“My whole thought process with having cancer is I will…I am open for anything as long as it will give me more time. As long as I didn’t have severe—very severe—pain, and I want to be around and help anyone else that I can. And what’s the best decision for me as an individual, and, uh, the decision we came to was the right one. And that spills over into the way Dr. [Name] is and all…I really feel that they’re looking at me. I don’t feel like a number.”
Emotional coping	“A 70 percent reduction. I was elated. I was happy. I had no side effects, and I couldn’t have been happier. My Doctor [Name] was very happy. Then what happened was I had a scan and the scan came back saying that I had one or two new nodes that were somewhat active in my neck as I recall. The protocol of the trial says any new nodes, you know, you come off the trial. Well, that’s where the protocol of the trial and the patient benefit collided, because I’m obviously benefiting if their own records show that I had a 70 percent reduction and I had no side effects and no nodes that I could feel. They were all so small that they were internal type things that you couldn’t feel.” “Yes, I was scared because I also knew that I was no longer gonna be part of the clinical trial. But I just didn’t know what it meant for me at that time.”
Disappointment	“I haven’t gotten back to feeling good and normal…since [CCT withdrawal]. So, you know, that didn’t make me feel good. But the other side is, okay, but, you know, hopefully…this was my one chance to maybe prevent the lymphoma from coming back and now that option’s off the table. So now I have to deal with…if it comes back, I gotta deal with whatever…do chemo or whatever the heck the next step is if this does come back. So, I guess my safety net is gone. I don’t have that. So that’s kind of disappointing that if there was an option…to potentially keep this from coming back, that’s probably gone.”
Support system	“I’ve really been blessed. I mean, that’s really the best way I can put it, I just feel like I’m so fortunate, and I thank god that I have the team around me and family, friends, the social network.”
Burden of adverse effects	“No, [CCT withdrawal] wasn’t a hard thing because, you know, not being able to breathe, it really takes you aback, you know. You sit there and even though you’re just sitting, you’re trying to catch your breath, knowing that the drug caused this. It was not a difficult decision.” “And for this particular trial—and I know this is not true for most clinical trials—the new technology that I was talking to you about could be considered to be torturous in nature. It invoked tremendous pain and it was a characteristic of the treatment, and therefore I could see people who had other issues with the trial dropping out, given that fact. I mean if you add in particularly a difficult treatment with administrative considerations and other things, I can see where people would drop out of the trial or might not recommend the trial to others.”
Professional trust and support	“I truly am impressed, pleased, and even overwhelmed with the quality of care I got from [Hospital].” “I mean everybody from the guy who tells you to park to the doctor, the surgeons or whatever, they’re all on the same level. They’re very friendly and comforting, and they make you feel at ease there.” “I realize how busy these people are and…and it’s not that you’re a number, but you don’t have that particular closeness that you would have if you were speaking to your oncologist who had been seeing you for a while. You know?” “Sometimes [withdrawal] was just a shut off and don’t even worry about it. I think I got a little bit more of that from Dr. [Name], and I don’t mean to slam him. I deeply felt the highest regard for him when I first met him, but I just…as the times went down and more people got involved, I felt like he was not forthcoming with information.” “It just seems to me that had the drug people in their protocol been a little bit more open-minded and if that could be incorporated in some way, which is probably an impossibility because you’re dealing with so many drug companies and to try to get them all to do this is...it just seems like if they would be a little bit more open-minded to patient benefits as opposed strict protocol.”

Abbreviations: CAT, computer-aided tomography; CCT, cancer clinical trial; PE, pulmonary embolism.

### Post-CCT Prognostic Awareness

Patient-participants described their CCT withdrawal experience in multiple ways. Many characterized withdrawals as a failure of the trial drug. Others blamed their cancer for growing despite treatment or blamed the CCT’s protocolized nature, pledging allegiance to their oncologist. Others were unclear why they were removed and voiced assumptions about reasons. Finally, some described the decision as jointly made with their oncologist. Several subthemes highlight the range of participants’ post-CCT prognostic awareness, including where the patient is now; regrets, hindsight, and should-haves; and urgency to start the next treatment. At the beginning of each interview, a general “How are you doing?” question was asked. Some participants reported a positive outlook despite treatment failure, others admitted to feeling horrible, and 1 revealed she was in hospice care after the CCT.

Participants rarely used the medicalized language of *withdraw*, but instead used verbs such as *taken off*, *removed*, or *stopped*. Some disagreed with the research team’s decision to remove them or stated they had no choice in the decision. After withdrawal, some expressed regret at having enrolled in the trial. Many wondered whether enrollment had been a wise decision because it interfered with living their life. Others speculated there might be another drug that must work better for their type of cancer and regretted not choosing such a medication, instead of enrolling in the CCT.

For those with a good understanding of advanced-stage cancer, there was a sense of urgency to start the next treatment after CCT withdrawal, which stemmed from the knowledge that their cancer would continue to grow without treatment. Some participants enrolled in the next trial at the same appointment during which they learned they had been withdrawn from the original CCT. Many participants (15 of 20 participants [75.0%]) expressed an unwavering belief that CCTs are the source of breakthrough treatments and wanted to enroll in another trial.

### Goals of Care Discussions

When reflecting on CCT withdrawal, participants also considered their cancer journey and its emotional roller coaster. These goals of care discussions included reflections around decision-making, living with cancer, other options (or lack thereof), values, advanced care planning, acceptable trade-offs, hopes, the odds of effectiveness or survival, and personhood. More than two-thirds (13 of 19 participants [68.4%]) of patient-participants had an advance directive. Some reflected on their decision to enroll in a CCT as part of their value system, which shaped their care goals. Values shaping care goals included the importance of having more time to pursue life goals, doing any treatment vs no treatment, and living to continue to care for their family or children. Also reflected in comments about care goals were implicit fears. For many (14 of 20 participants [70.0%]), the ultimate fear was death, and they bargained for more time. For them, CCTs represented hope or the key to survival.

In making a decision based on personal goals, patient-participants weighed the benefits and burdens of enrolling. Some focused on numbers when making decisions. Others considered acceptable states of living or trade-offs (ie, survival time vs pain) when weighing options. Several (7 of 20 participants [35.0%]) valued their personhood and wished to be treated as more than their disease—that is, more than just a number or a trial participant. Feeling valued as a person creates a therapeutic relationship and can support the decision-making process.

### Emotional Coping

Patient-participants reported intense emotions throughout their journey, starting with the diagnosis. CCT participation and withdrawal generated many emotions: hope, fear, sadness, anxiety, shock, and even happiness. Some described feeling emotionally vulnerable, even naked, as a result of seeing a new oncologist, differences in monitoring procedures, or the uncertainty of it all. Some described certain treatments, including CCTs, as a safety net, which contributed to a sense of hope. After CCT withdrawal, they reported experiencing disappointment and a loss of hope. One said that she feared the unknown and what CCT withdrawal meant for her cancer and her life.

Participants reported coping with fears and disappointment in various ways. Many (18 of 20 participants [90.0%]) discussed relying on personal and professional supports, including spouses, children, siblings, friends, neighbors, colleagues, medical staff, and others with cancer, to support them following CCT withdrawal. Although some took on the burden of decision-making as solely their own, all expressed support from a social network, which they linked to their emotional coping.

### Burden of Adverse Effects

Five of 20 patient-participants (25.0%) reported withdrawing from the CCT because they were experiencing unbearable symptoms from the treatment, including fatigue, pain, dyspnea, neuropathy, chills, coughing, diarrhea, hives or rash, blindness, and loss of hearing. Many of these symptoms had a negative effect on their daily lives. When patient-participants and their physicians believed the burden outweighed any benefits, they were removed from the CCT. Others experienced symptoms only at the time of administration of the CCT drug.

### Professional Trust and Support

When reflecting on their trial withdrawal, patient-participants often discussed their medical practitioner, research team, and/or overall impressions of the research center. Positive reflections focused on the contribution of nursing care to their trial experience, their trust and respect for their oncologist, or the professionalism and caring nature of staff. However, some noted a lack of professionalism among staff. One was disappointed with the impersonal quality of his experiences with the researcher oncologist, compared with a local oncologist with whom he had a closer relationship. Others had mixed feelings toward the medical staff, noting both positive and negative interactions. For example, one expressed disappointment with a physician’s communication about the CCT withdrawal decision while continuing to hold much respect for the researcher.

Some patient-participants thought the withdrawal decision was dictated by too many fixed rules and rigid protocols. When they were removed because of protocols and drug developer rules, they thought this took away both the physician’s clinical judgment and the individualized, patient-centered care that they deserved.

## Discussion

To our knowledge, this study is one of the first to focus on understanding the perspectives and experiences of patient-participants when they withdraw voluntarily or involuntarily from CCTs. This understudied area of research requires ethical attention, and our results will help identify what is needed for responsible transitioning of patient-participants from a CCT. We highlight 3 points for discussion. First, patient-participants transitioning out of a CCT described feeling intense symptoms and emotions and awareness that their life span was short and options seemed limited. Indeed, what happens at trial exit is just as important as what happens at entry, as many patient-participants were facing life-limiting illness. Second, limited discussions with patient-participants about their immediate posttrial care needs left many feeling that there was no clear path forward. Third, good communication that deliberatively includes attention to posttrial needs throughout the CCT is needed to help scared and disappointed patient-participants navigate the next steps.

As patient-participants expressed, having to withdraw from a CCT for whatever reason triggers many emotions, including sadness, fear, and hope for something else, as well as mixed emotions. A care transition is generally described as a transfer between different locations, practitioners, or levels of care.^4,13,14^ Effective transitions include communication about a care plan that incorporates the patient’s goals and preferences (including advanced care planning) and preparation for next steps. Most of the focus on post-CCT clinical care has been on providing patient-participants with access to any benefits that result from the trial, as affirmed in the Declaration of Helsinki.^15^ Cho et al^16^ argued that there is a broader set of posttrial responsibilities that have been overlooked by the research community. Needs related to posttrial care or responsible transitioning of participants are not well understood. After withdrawing from a CCT, patient-participants have a variety of needs, as our findings indicate. They may have a need for advanced care planning or end-of-life discussions or may want to discuss alternative interventions to the investigational drug or entry into another CCT. Many may need access to medical services and other community resources, as well as other supports. CCT patient-participants may experience many financial, emotional, physical, and spiritual hardships that can affect their health outcomes, as our data indicate.^17,18^ Our patient-participants were at different stages of their cancer trajectory, with some needing discussions of goals of care, some searching for new trials following their withdrawal, and others experiencing adverse effects. Although ineffective care transitions have mostly focused on older adults with chronic illnesses and their costly rehospitalizations, CCT patient-participants also need coordinated transitional care management, because they may need multiple treatments and specialists to support their ongoing health regimen.

Cho et al^16^ further argued that, on the basis of the principles of ethical research, every research participant should be offered posttrial care to varying degrees at trial exit. Posttrial care was not evident in our study, as most participants expressed that they had no other options than to participate in the CCT, and some were regretful about participation. In fact, one-half of our patient-participants (10 patients) had stage IV disease at cancer diagnosis, and 11 had stage IV disease by the time they enrolled in their CCT. Although more than two-thirds of our withdrawn patient-participants had an advance directive, we do not know whether there were any discussions or preparation for next steps. It could be that given their stage at diagnosis, goals of care discussions with their oncologist or others had occurred before CCT enrollment. These conversations should be ongoing throughout the trial, and we need more data to understand these types of discussions.

Patients with cancer experience many different symptoms depending on their type of cancer and treatment course. Indeed, these symptoms can be both physically and emotionally distressing, as our findings indicate. Symptoms such as pain, fatigue, and neuropathies can interfere with day-to-day functioning and outcomes of care.^19,20^ The patient-participants in our sample described a variety of distressing symptoms, including fatigue and respiratory, neurological, muscular, and gastrointestinal symptoms, consistent with other research.^21,22^ Given the distress participants were experiencing at CCT exit, it remains unclear whether palliative care consultation was offered at any point during the CCT. Furthermore, most patient-participants were withdrawn because of disease progression; at this stage of advanced cancer, referral to palliative care might be considered late along the cancer journey, but it should still be considered. It is interesting that patient-participants rarely acknowledged that disease progression was the reason for withdrawal. It may have been easier for them to consider reasons other than worsening disease and, therefore, worsening prognosis.

Patients with cancer may avoid palliative care as they continue to search for a cure or remission.^23,24^ In our study, this usually meant seeking other CCTs. Delivery of palliative care concurrently with disease-directed therapies should become a standard at cancer institutions, as palliative care consultation has been shown to improve quality of life and emotional states in patients with advanced cancer.^25^ LeBlanc^26^ proposed that palliative care specialists be considered part of the cancer team at CCT enrollment, especially for patients in early-phase trials. This approach has the added benefit of providing needed support to discuss goals of care and ongoing concerns throughout the trial.

Several participants left the CCT because of symptom burden. We do not know whether symptom burden increased at CCT exit because of the stress of withdrawing. Some evidence suggests that the experience of symptom burden may be affected by social, socioeconomic, and supportive factors,^27,28^ along with perceived stress and optimistic views.^29,30^ CCT patient-participants are generally very hopeful at trial entry, but hopefulness may wax and wane over the CCT’s course. More research is needed to measure hope over time in relationship to symptom burden at trial exit and beyond.

Finally, improving communication with the CCT research team is imperative as we help patient-participants transition and assess posttrial care needs. We concur with Lawton et al^31^ who noted that, “Despite the emphasis placed on treating trial participants in fair and ethical ways, it is noteworthy that these individuals have rarely been consulted about the care and support they feel they need at the end of a trial.” As one of our patient-participants noted, the whole experience was a roller coaster. Members of clinical and research teams should more deliberately discuss what responsible CCT withdrawals should include, types of discussions that are needed, timing of such discussions, and who should begin these discussions with patient-participants and families. The emotional bonds that patient-participants build with their research team are often broken at CCT exit. The transition to new practitioners also needs attention. Some authors have indicated that loss of contact with accustomed practitioners can be a form of emotional harm for patient-participants, who may experience bereavement through this loss.^32,33^

### Limitations

Our study was limited in several ways. First, we recruited patients from a single cancer institution in 1 geographical area. Patient-participants may view their withdrawals differently from those at other cancer institutions depending on whether they received support for their symptom burden and posttrial care needs. In addition, their views on what is owed them may differ depending on their trial.^34^ Second, although we identified some ethical challenges arising at CCT exit, more work is needed to explain these issues from a diverse group of patients with cancer, including the emotional, physical, and cultural influences that might affect posttrial needs. Third, although the sample size was appropriate for qualitative research, we cannot generalize beyond the study sample, and larger quantitative samples may be helpful to test hypotheses and factors associated with CCT withdrawal.

## Conclusions

Participating in CCTs affords an opportunity for novel and experimental therapies that may extend life and, in some instances, may even cure the disease. CCT participation involves risks that may prove to outweigh benefits. Exiting a CCT represents a type of risk for participants, especially those withdrawn because of disease progression, lack of effect, toxic effects, or symptom burden. They must navigate next steps, including end-of-life care. CCT team members should do all they can to help patients at exit from CCTs, and this requires more dialogue and research from all stakeholders to identify best practices. Understanding the posttrial needs of patients with cancer and their families and what constitutes responsible transitions represents a measure of ethical respect for the many contributions that patients with cancer make to advancing our scientific knowledge and finding treatments that save lives.
