# Error in Funding/Support Statement

**DOI:** 10.1001/jamanetworkopen.2021.29286

**Published:** 2021-09-03

In the Original Investigation titled “Experiences of Patients After Withdrawal From Cancer Clinical Trials,”^1^ published August 10, 2021, there was an error in the Funding/Support statement at the end of the article. It should have stated that grant T32NR009356 to Ms Foxwell was from the National Institutes of Health. This article has been corrected.^1^
